# IgE as a predictor to omalizumab response in patients with chronic spontaneous urticaria

**DOI:** 10.3389/falgy.2024.1451296

**Published:** 2025-01-23

**Authors:** Luis Felipe Ensina, Larissa Brandão, Luisa Karla Arruda, Faradiba Sarquis Serpa, Régis Albuquerque Campos, Solange Rodrigues Oliveira Valle, Paulo Ricardo Criado, Sarbjit Singh Saini, Roberta Fachini Jardim Criado

**Affiliations:** ^1^ Department of Pediatrics, Federal University of São Paulo, São Paulo, Brazil; ^2^ Ribeirão Preto Medical School, University of São Paulo, Ribeirão Preto, Brazil; ^3^ Division of Allergy and Immunology, Escola Superior de Ciências da Santa Casa de Misericordia de Vitória, Vitória, Brazil; ^4^ Department of Internal Medicine, Federal University of Bahia Medical School, Salvador, Brazil; ^5^ Department of Internal Medicine, Federal University of Rio de Janeiro, Rio de Janeiro, Brazil; ^6^ Department of Dermatology, Centro Universitário Faculdade de Medicina do ABC, Santo André, Brazil; ^7^ Division of Allergy and Clinical Immunology, The John Hopkins School of Medicine, Baltimore, MD, United States

**Keywords:** urticaria, immunoglobulin E, omalizumab, biomarkers, treatment

## Abstract

This multicenter study aimed to explore whether baseline total immunoglobulin E (IgE) levels could predict omalizumab response in chronic spontaneous urticaria (CSU) patients. Refractory CSU patients, treated with omalizumab after failing second-generation H1-antihistamines, were analyzed retrospectively across seven centers in Brazil. The study assessed total IgE levels at baseline, comparing responders to non-responders and considering complete and partial responses. The results showed a significant reduction in CSU symptoms post-treatment. Non-responders had lower baseline IgE levels. A sensitivity of 67.8% and specificity of 93.3% for predicting a response were found at an IgE level of 59.5 IU/ml. Similar values were observed for complete responders. Notably, a baseline IgE level lower than 59.5 IU/ml may indicate late responders. The study underscores the potential of baseline IgE levels as a predictive biomarker for omalizumab response in CSU patients. Further research, incorporating diverse populations and analyzing response variables, is warranted to validate these findings.

## Background

Total immunoglobulin E (tIgE) serum levels have emerged as a dependable biomarker for predicting omalizumab response in chronic spontaneous urticaria (CSU) patients (1). However, challenges persist in establishing standardized cutoff values, sensitivity, and specificity, as variations arise across diverse populations and methodologies.

The aim of this multicenter study was to investigate whether baseline levels of total IgE can serve as a predictive indicator of response to omalizumab in CSU.

## Methods

In this multicenter retrospective analysis of a CSU patient registry, we evaluated the clinical findings and total IgE levels of patients who were refractory to second-generation (SG) H1-antihistamines and were treated with omalizumab in seven Urticaria Centers of Reference and Excellence (UCARE) centers in Brazil.

Patients with a diagnosis of chronic urticaria based on their clinical history and the presence of clinical signs and symptoms were included in the registry. For this analysis, we excluded patients with isolated chronic inducible urticaria.

Refractoriness to H1-antihistamines was evaluated after a minimum 4-week course of a fourfold dosage of an SG non-sedating H1-antihistamine (e.g., bilastine, levocetirizine, fexofenadine, desloratadine, and loratadine). For these patients, treatment with 300 mg of omalizumab every 4 weeks was prescribed. Patients were instructed to maintain the high dosage of the SG H1-antihistamine until achieving complete symptom control. Response to omalizumab was evaluated at week 24 and was considered complete [complete responders (CRs)] for patients with an urticaria activity score in 7 days (UAS7) of 0 or an urticaria control test (UCT) score of 16. Partial responders (PRs) were those with a decrease in their UAS7 of ≥10 or an increase in their UCT score of ≥3 points from baseline (2).

tIgE serum levels were measured using an immunofluorimetric assay (ImmunoCAP™; Thermofisher, Uppsala, Sweden) at baseline, before starting omalizumab treatment, and were expressed in IU/ml. The study analyzed and compared the median IgE levels among responders and non-responders (NRs) and among CR and NR. An receiver-operating characteristic curve (ROC) analysis was performed to define the cutoff points for the sensitivity and specificity of tIgE and response to omalizumab.

The study protocol was approved by the ethics committee of the Federal University of São Paulo. All patients who agreed to participate in the registry provided informed consent.

## Results

The complete registry comprises 265 patients with chronic urticaria treated with omalizumab from July 2017 to April 2023. We included 158 patients in the study who had accessible baseline tIgE data, of whom 84.8% were women and the mean age was 41.9 years (ranging from 7 to 83). Patients without baseline IgE data were excluded from the analysis (*n* = 107), of whom 76% were women with a mean age of 44.7 years (ranging from 13 to 83).

According to prior studies and our previous findings, omalizumab significantly reduced CSU symptoms in most of the patients. After 24 weeks of treatment, 75.9% of patients were CRs, 14.6% PRs, and 9.5% NRs.

Baseline median tIgE levels were significantly lower in the NR group when compared to the responder group (including partial responders) and the only CR group (Table 1).

**Table 1:** Omalizumab response at 24 weeks and baseline IgE levels.

	Response in 24 weeks		
Variables	Yes (*n* = 123)	No (*n* = 15)	*p*-value^a^
	Median (P_25_; P_75_)	Median (P_25_; P_75_)	
IgE	111,0 (43,0; 296,0)	19,0 (6,0; 54,0)	< 0,001
	Complete response in 24 weeks		
Variables	Yes (*n* = 120)	No (*n* = 15)	*p*-value^a^
	Median (P_25_; P_75_)	Median (P_25_; P_75_)	
IgE	135,5 (48,3; 300,5)	19,0 (6,0; 54,0)	< 0,001

^a^ Mann-Whitney Test.

As determined by ROC analyses, a tIgE level of 59.5 IU/ml has a sensitivity of 67.8% and specificity of 93.3% to predict a response to omalizumab. When considering only the complete responders, an IgE level of 59.5 IU/ml has a sensitivity and specificity of 70.8% and 93.3%, respectively. The cutoff values were similar when analyzing adults and older patients (≥60 years). Due to the low number of children treated, it was not possible to analyze this subgroup of patients.

## Discussion

Our results confirm earlier real-life studies that demonstrated that omalizumab is effective in CSU, completely controlling the disease in 67.9% of patients after 24 weeks of treatment (3).

While Metz et al. and Viswanathan et al. did not find significant differences in serum IgE levels between omalizumab responders and NRs, Ertas et al. reported that NRs to omalizumab had lower baseline IgE levels compared to CRs after 4 weeks of treatment (17.9 vs. 73.7 IU/ml) (4–6). They also demonstrated that patients with IgE levels lower than 43 IU/ml had a 33% risk of non-response within the first 12 weeks of treatment, compared to just 5% in patients with IgE levels higher than 43 IU/ml (6). Similar findings have been confirmed by other authors (7–9).

Straesser et al. reviewed the charts of 137 patients older than 12 years of age with refractory CSU and measured baseline IgE (bIgE) levels, subdividing IgE levels into subquartiles. Patients with a total IgE level under 15.2 IU/ml had a 48.4% response rate, compared with more than 86% for patients with levels of 15.3 IU/ml and greater (10).

In our study, it is noteworthy that when considering complete responders and a baseline IgE level of 42.5 IU/ml, the test demonstrated a sensitivity of 76.7% and a specificity of 73.3% for predicting a response. However, for a higher specificity (93%) and a sensitivity of 70.8%, we should consider IgE levels of 59.5 IU/ml.

We decided to evaluate our patients at 24 weeks due to possible late responders. In this context, a recent meta-analysis indicated no significant difference in baseline serum tIgE levels between CRs and PRs within 12 or at 24 weeks in a subgroup analysis for timing of outcome. However, CRs to omalizumab within 4 weeks after the first dose had significantly higher serum total IgE levels compared to late CRs (7).

In another comparative study between omalizumab late responders and non-responders, the total IgE levels were below 50 UI/ml in 77% of patients with an equal distribution between both groups, with median total IgE levels of 42 UI/ml in late responders and 9 UI/ml in non-responders (11). These data suggest that a baseline IgE level lower than 59.5 IU/ml should be considered for late responders. However, in this study, we did not separately analyze early and late responders.

Finally, in a previous study in Brazil, the authors found that the tIgE level was higher than 100 IU/ml in 82 of 267 patients (32.2%) with CSU, supporting a considerable chance for successful omalizumab treatment in Brazilian patients with CSU, as was observed in this study (12).

Some limitations of this study include its reliance on a patient registry, which may result in critical data being missing. For instance, baseline IgE levels were unavailable for 40% of the patients, leading to their exclusion from the analysis and potentially influencing the results. In addition, data on total IgE levels at week 4 were not collected. This is a notable limitation, as recent studies suggest that the ratio of IgE level at week 4 to baseline IgE level is a more sensitive predictor of omalizumab response, particularly in explaining why some patients with low baseline IgE levels still respond to treatment (6, 13).

In conclusion, to the best of our knowledge, this is the first multicenter study emphasizing the role of baseline IgE levels as a potential predictive biomarker for omalizumab response in specific populations. A cutoff value of 59.5 IU/ml is valid for the Brazilian population but may not exhibit the same sensitivity and specificity for other populations. Future studies involving centers in different countries and deeper analysis considering variables such as time to response and age groups are still needed.

## Data Availability

The raw data supporting the conclusions of this article will be made available by the authors, without undue reservation.
